# Development of a Rapid Surveillance System for Ross River Virus in Mosquitoes Through Reverse-Transcription Loop-Mediated Isothermal Amplification (RT-LAMP)

**DOI:** 10.1155/tbed/1772438

**Published:** 2025-02-28

**Authors:** Alexandra Knox, Gemma Zerna, Travis Beddoe

**Affiliations:** Department of Animal, Plant and Soil Sciences, Centre for AgriBioscience La Trobe University, Bundoora 3082, Victoria, Australia

## Abstract

The global rise in arboviral diseases can be attributed to the ongoing effects of climate change. Ross River virus (RRV) is an illustrative example of such diseases, with case reports in Australia experiencing a significant surge since 2020. RRV is transmitted to susceptible species, such as horses and humans, through multiple mosquito vectors, namely *Culex annulirostris*, *Aedes camptorhynchus*, and more recently *Ae. notoscriptus*. This disease is not only endemic to Australia but has caused outbreaks in surrounding countries such as Fiji and Papua New Guinea. Currently, there are no therapeutic regimes or vaccinations available for RRV, leaving public health warning systems and advice relying upon disease prediction and surveillance. Commonly utilised methods, such as predictive modelling, are experiencing challenges resulting from an increased mosquito presence and extreme weather patterns, often yielding inaccurate advice. Reverse-transcription quantitative polymerase chain reaction (RT-qPCR) provided a promising solution to mitigate these challenges and is now considered the gold standard in many Australian states. However, this method must be performed in a laboratory setting and requires expensive machinery, thus rendering it inadequate for resource-poor or rural communities. Reverse-transcription loop-mediated isothermal amplification (RT-LAMP) serves as a simple and field-deployable substitute with comparable sensitivities and specificity to RT-qPCR, whilst possessing the ability to provide rapid results within 20 min. This paper describes a novel RRV RT-LAMP assay that can detect RRV in as little as one mosquito, with a limit of detection of 1 × 10^−7^ ng/µl (~620 copies/µl) and a clinical sensitivity of 84%. Through the addition of tetramethylammonium chloride (TMAC), our assay achieved a 100% specificity and was able to detect RRV RNA as early as 2 min in crude field samples. The simplistic sampling method coupled with our RRV RT-LAMP assay can provide an in-field and low-cost alternative to current routine surveillance techniques.

## 1. Introduction

Ross River virus (RRV) is the most frequently reported arbovirus in Australia, causing infections in both humans and horses [1, 2]. The economic impact of this virus amounts to a conservative estimate of $AUD 4–5 million annually, with additional expenditures allocated to control programs [3, 4]. Infections are not limited to Australia as endemic and enzootic outbreaks also occur in surrounding regions, such as Papua New Guinea and the South Pacific Islands [4, 5]. As the virus spreads through mosquito bites, various mosquito species have been identified as vectors of RRV in Australia. Notably, *Culex annulirostris*, *Aedes vigilax*, and *Ae*. *camptorhynchus* are considered as the primary vectors; however, *Ae*. *notoscriptus* has recently emerged as a vector in urbanised areas [6–8]. This range of viable vector mosquito species has been attributed to RRV's spread and adaption to the diverse environments in Australia, consequently resulting in an increase in reported cases [2, 8].

It is well established that RRV-infected humans can experience debilitating arthritogenic illness which can become long-term [1, 9]. Conversely, the susceptibility of horses to RRV was initially a matter of contention in the literature, due to the non-specific disease presentation during infection. Nevertheless, multiple studies now agree that clinical manifestation in horses includes lethargy, poor performance, muscle pain and stiffness, ataxia, and limb oedema [10, 11]. Due to these ambiguous symptoms, a diagnosis of RRV cannot be determined through examination of clinical signs alone in horses and thus requires additional serological testing [2, 10]. In humans, a definitive diagnosis of RRV requires both nucleic acid testing through reverse-transcription polymerase chain reaction (RT-PCR) as well as demonstration of immunoglobulin (Ig) seroconversion, from IgM to IgG [2, 12]. Moreover, there are no specific therapeutic treatments for RRV recovery or protective vaccines for both susceptible species, therefore, a robust surveillance system is paramount for protection [1, 2].

Currently, the Australian government is dependent on predictive computational modelling and mosquito surveillance methods, such as reverse-transcription quantitative polymerase chain reaction (RT-qPCR), as an early warning system for potential RRV outbreaks [13–15]. However, due to the unique ecology of RRV's transmission cycle, which has the potential to encompass a range of mosquito vector species and vertebrates, alongside environmental influences, the surveillance, prediction, and mitigation of the disease has proven difficult in recent years. Additionally, RRV's complex spatiotemporal nature impedes predictive modelling, often resulting in inaccurate advice of disease activity [14–16]. Due to climate change mosquito populations are becoming more prevalent, attributed to prolonged mating periods from high temperatures, and expansion of breeding habitats resulting from extreme weather events [17, 18]. For example, droughts seen throughout Australia have resulted in mosquitoes migrating to coastal and urbanised regions in search of breeding habitats. A study conducted in 2013 by Trewin et al. [19] noted a substantial increase of *Ae*. *notoscriptus*, a “container-breeding” mosquito species, during a severe drought in Brisbane, Australia. In response to the drought, the local governments in Queensland provided over 21,000 households with rebates on collected rainwater, subsequently providing an increase in water storage, which in turn resulted in ample breeding grounds for this mosquito species [19]. Additionally, recent unrelenting flooding has exacerbated optimal breeding habitats for mosquitoes, further stimulating population growth. The expansion of mosquito populations has inevitably resulted in a recent increase of RRV cases reported across the country [18, 20, 21].

Spikes in cases have further been driven by the urbanisation of inland and coastal regions of Australia, bringing human populations closer to RRV reservoir hosts, such as kangaroos and wallabies [22]. However, outbreaks occurring in neighbouring countries lacking these species indicated that reservoir hosts of RRV extend beyond the previously assumed macropods and marsupials. Recent studies have identified a range of host species, encompassing both domesticated and wild animals such as canines, cattle, goats, as well as rodents and passerine birds [22–24]. For example, between 1979 and 1980, Fiji experienced an epidemic outbreak at which time reported cases exceeded 500,000, with some regions experiencing a 90% seroprevalence in humans post-outbreak [25]. As Fiji has no resident populations of macropods or marsupials, it was theorised the outbreak was driven via an urban transmission cycle, involving only mosquitoes and infected or susceptible humans. However, as Fiji is still experiencing a continuous, now endemic, circulation of RRV, further research has revealed there is a current seropositivity of 48% found in domestic animals [24].

With this expansion of reservoir hosts, it is evident that surveillance techniques must continue to focus on mosquito monitoring systems. Concurrently with mosquito presence increasing, it is apparent these programs will require expansion, applying greater pressure on already stretched systems. While the use of predictive modelling can be an insightful tool for forecasting potential outbreaks and produce valuable public health advice, they do not provide an exact statistic [14–16]. Accordingly, mosquito surveillance remains the most accurate technique for informed advice and action. Current arbovirus surveillance systems are typically conducted via viral isolation or RT-qPCR [13, 26–28]. Whilst these tests show high sensitivity and specificity, they both rely on expensive machinery and trained personnel, thus requiring well-equipped laboratories. Additionally, viral isolation is laborious and can take up to a week to obtain results, causing significant delays between sample collection and results, thus hindering timely intervening protocols to limit transmission. Conversely, RT-qPCR can provide same-day results. However, stringent and lengthy viral extraction and purification protocols are needed due to inhibitors in environmental and tissue samples [29, 30].

To overcome these limitations of traditional surveillance techniques, Notomi et al. [31] developed an isothermal amplification method for detection of nucleic acid, termed loop-mediated isothermal amplification (LAMP). As these assays only require a single constant temperature, LAMP can be performed using any heat source, negating the need for expensive machinery. LAMP is a highly rapid and sensitive DNA amplification technique that employs a strand-displacing polymerase and 4–6 primers to target various areas of a gene of interest and provides results within 20 min [29, 31]. Furthermore, the LAMP technique has expanded to integrate reverse-transcription (RT-LAMP), allowing for the conversion of RNA to cDNA within the same reaction as DNA amplification. This simultaneous activity of RT alongside a strand-displacement DNA polymerase eliminates the need for additional steps conventionally required for RT, thereby presenting a rapid alternative to the detection of RNA. Both LAMP and RT-LAMP are designed to be utilised in-field, which greatly reduces the time between sample collection, results, and ultimately biosecurity implementation [32]. Additionally, these assays are highly tolerable of typical inhibitors found in samples without compromising the integrity of the assay [32, 33]. Currently, RT-LAMP has been developed and utilised for a range of viral surveillance programs, including Zika virus [34], Ebola virus [35], St Louis and Western Equine encephalitis, and West Nile virus [36]. These assays continuously prove to be reliable technologies for an array of environments, making them ideal for RRV surveillance in Australia's diverse territories.

Herein, we present a novel RRV RT-LAMP assay developed as an improved monitoring system for detection in mosquitoes. A synthetic positive control was designed for this assay to enable optimisation and subsequent validation with field sample testing. Our assay, coupled with a simple “one-tube” RNA extraction procedure, has allowed for a rapid surveillance program that can monitor results in real-time.

## 2. Materials and Methods

### 2.1. RT-LAMP Primer Design

Primers were developed to target a conserved region of RRV envelop protein 2 (E2) gene (accession no: KX709870.1) (Table 1). Previous studies comparing closely related alphaviruses, Barmah Forest virus (BFV), Semliki Forest virus (SFV), and Sindbis virus (SINV), revealed a unique pinpoint mutation in RRV within the E2 gene [37]. Primers were designed to cover the area of deviation from other alphavirus using online software PrimerExplorer (V5, Eiken Chemical Co., Ltd., Tokyo, Japan) (Figure S1) and ordered through Bioneer Pacific (Daejeon, South Korea).

### 2.2. Preparation of Synthetic RNA Positive Control

**Figure 1:**
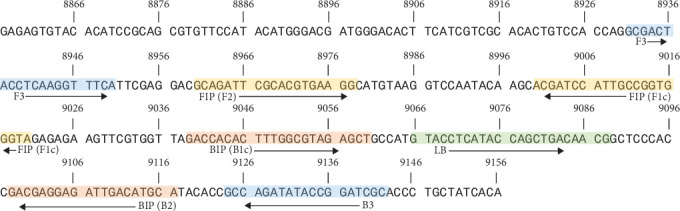
The designed 300 bp RRV synthetic positive control sequence containing the partial E2 gene. Positions of the RRV RT-LAMP primers are highlighted throughout. The forward outer primer (F3) and backward outer primer (B3) are shown in blue, forward inner primer (F2 and F1c) are shown in yellow, backwards inner primer (B2, B1c) are shown in orange, and the backward loop (LB) is shown in green. Genome positioning according to GeneBank accession no: MH987780.1. RRV, Ross River virus.

The preparation of the synthetic positive control was partially based on a previously published article for foot and mouth disease [38]. Briefly, the synthetic positive control was designed and ordered as a 300 bp dsDNA plasmid containing a segment of the E2 gene where the primers overlapped (Figure 1), through Bioneer Pacific into the standard cloning vector pBHA (Bioneer Pacific). The plasmid was amplified via PCR using the RRV LAMP's forward outer primer (RRVE2_F3) modified with a T7 promoter sequence (underlined below) at the 5′ end (TAATACGACTCACTATAGGCGACTACCTCAAGGTTTCA), and the backwards outer primer (RRVE2_B3) (Table 1) as the reverse primer, to produce a 212 bp product. Reactions contained 0.8 µM of each primer, 50 µl of Go-Taq Green mastermix (Promega, Wisconsin, United States), and 20 ng/µl of the synthetic positive control, adjusted to a final volume of 100 µl with dH_2_O. Thermocycling was performed under the following conditions: 95°C for 5 min, 40 cycles of 95°C for 1 min, 54°C for 1 min, and 72°C for 2 min, followed by a single cycle of 72°C for 5 min. Products were resolved on a 2% ethidium bromide (0.5 µg/ml) agarose gel for 40 min at 100 amps, followed by PCR product purification using NucleoSpin PCR Clean-Up Kit (Takara Bio, Inc., Shiga, Japan). The purified products (1 µg/µl) then underwent transcription to RNA via T7 DuraScribe Transcription Kit (Lucigen Corp, Wisconsin, United States) following the manufacturer's guidelines with a 6-h incubation period. The RNA was then purified with 1 µg/µl of glycogen (Product code: N632-2X0.5ML, VWR Life Science, Pennsylvania, United States) following the manufacturer's guidelines. The final RNA product was resuspended in 20 µl of dH_2_O and stored at −20°C prior to use as a template for the RRV RT-LAMP assay optimisation and validation.

### 2.3. Optimisation of the RRV RT-LAMP Assay

To determine the optimal primer concentrations for the RRV RT-LAMP assay, a series of tests were conducted with a range of concentrations for FIP, BIP, and backward loop (LB), using the RNA synthetic positive control as a template. Each concentration was performed in triplicates in 25 µl reactions containing 15 µl of ISO-DR004-RT master mix (OptiGene Limited, Horsham, United Kingdom), 0.2 µM of F3 and B3 primers, 0.8–1.6 µM of FIP and BIP, and 0.4–1.0 µM of LB, with 1 ng/µl of the synthetic positive control, or 5 µl of dH_2_O for a no template control (NTC). The assay was performed with real-time results monitoring fluorescence outputs using the Genie II machine (OptiGene Limited), with amplification at 65°C for 30 min, followed by an annealing temperature of 98°C to 80°C, with a ramping rate of 0.05°C/s. Reactions that reached the fluorescence ratio threshold of 0.02 normalised fluorescence units prior to 20 min were considered successful, as per the manufacturer's guidelines (OptiGene Limited). Once optimal primer concentrations were determined, the assay was performed at amplification temperatures from 60°C to 66°C, followed by the same anneal temperature and ramp rate as stated. Results were considered positive if amplification occurred in all technical replicates (i.e., 3/3 amplified) under the same conditions as above.

### 2.4. RRV RT-LAMP Assay Analytical Performance and Optimisation With Tetramethylammonium Chloride (TMAC)

To test the specificity of the RRV RT-LAMP assay, plasmids of the E2 gene of closely related alphaviruses, BFV (GenBank accession no: L79899.1), SFV (GenBank accession no: X78112.1), and SINV (GenBank accession no: JX682560.1) were designed and prepared as per preparation of the synthetic positive control. Two separate screenings of specificity occurred, first using the optimal primer conditions and assay temperature, with triplicate technical replicates of 1 ng/µl of each RNA synthetic control per virus gene. Then, second, the same conditions with the addition of TMAC to suppress non-specific applications noted in the initial screening. TMAC was tested at set assay concentrations of 5, 10, 20, and 40 mM, against each RNA synthetic positive control. All assays were performed for 30 min to observe any amplification of non-target RNA.

The RRV RT-LAMP assay's limit of detection (LOD) was determined using the optimal primer concentrations with the addition of 20 mM of TMAC. The synthetic positive control was serially diluted 10-fold from 1 × 10^−1^ ng/µl (6.24 × 10^8^ copies/µl) to 1 × 10^−9^ ng/μl (6.24 copies/µl) in dH_2_O. Each serial dilution was assessed using triplicate technical replicates with each assay then being repeated five times. The LOD was defined as the lowest concentration of RNA which returned a positive result consistently in each sample before 20 min of amplification with a fluorescence ratio threshold of 0.02 normalised fluorescent units. The inter–coefficient of variation (CV) was determined as the average time to positive (Tp) of technical replicates divided by the standard deviation (SD) of each Tp per replicate, across five assays. Additionally, both the LOD and inter-CV were performed and calculated using the RRV RT-LAMP assay without the addition of TMAC to determine if there were any adverse effects on the assay in terms of LOD and Tp.

### 2.5. Mosquito Spiking Preparation and RNA Extraction

Mosquitoes were collected around the eastern suburbs of Victoria using carbon dioxide–baited surveillance traps from November to March in 2022–2023. Traps were set overnight and collected the following day. Mosquito vector species, namely *Ae. camptorhynchus*, *Ae. notoscriptus*, and *Cx. annulirostris*, were identified using taxonomic keys and stored at −20°C until use. To simulate the presence of RRV in the samples, the target synthetic gene sequence was added to collected mosquitos, in a mock spiking sample trial. Pools of ~10 mosquitoes were submerged into 100 µl of the RNA RRV synthetic positive control at 20 ng/µl, diluted in dH_2_O, for a theoretical final concentration of 0.1 ng/µl (1 × 10^7^ copies/µl) post-RNA extraction. The mosquitoes were then placed into fresh 1.5 ml tubes and dried for 5 min at room temperature then stored at −20°C until use.

Three RNA extraction methods were tested and evaluated using both spiked mosquitoes and non-spiked mosquitoes, to determine an appropriate technique for in-field surveillance. The first method was adapted from Lamb et al. [39], following the described “in-tube” protocol with an additional step. Briefly, ~10 mosquitoes were placed into a 1.5 ml tube and homogenised in 200 µl of phosphate-buffered saline (PBS:137 mM NaCl, 2.7 mM KCl, 10 mM Na_2_HPO_4_, 1.8 M KH_2_PO_4_, pH 7.4) using a P10 size pipette tip. A 1-min centrifuge step at 6000× *g* was added to aid in bulk sampling. Next, a simplistic “in-syringe” technique was evaluated with slight amendments from Bhadra et al. [40], where a 0.5 µM pore (1/8th frit) was inserted into a 1cc syringe with ~10 mosquitoes. After maceration with the syringe plunger against the frit, 200 µl of nuclease-free H_2_O was added to the syringe and filtered into a 1.5 ml tube. The last method was developed as a “one-tube” RNA extraction technique, similar to those described above. Here, 10 mosquitoes were placed in a 1.5 ml tube and homogenised using a G-tube polypropylene pestle in 200 µl of PBS. The sample was subsequently drawn into a 1 cc syringe and filtered through a 0.7 µM pore GD/X syringe filter (Merck KGaA, Darmstadt, Germany) back into the same tube. Each extracted sample was quantified using Qubit RNA High Sensitivity Kit (Thermo Fisher Scientific Pty Ltd., Massachusetts, United States), before being stored at −20°C until use. Each extraction method included three non-spiked mosquito pools as controls. To confirm the presence of the correct gene of interest, the spiked mosquitoes also underwent Sanger Sequencing through AGRF (Melbourne, Victoria) following PCR, as described above. Following confirmation, 5 µl of each crude sample was evaluated on the RRV RT-LAMP assay as described above, however with a fluorescence ratio threshold of 0.01 normalised fluorescence units. Each assay contained the RRV RNA synthetic positive control and an NTC, as well as three non-spiked mosquito pools for each extraction technique. Samples which amplified before 20 min were considered positive.

To determine a LOD on mosquito samples, spiked mosquitoes that were extracted using the “one-tube” method were serially diluted 10-fold (1:10, 1:100, 1:1,000, 1:10,000, and 1:100,000) in PBS. The dilute spiked samples were tested in duplicates on the RRV RT-LAMP assay as above. Additionally, mosquitoes were spiked in pools as described above with the RRV RNA synthetic positive control, and sequentially separated into groups of 5, 4, 3, 2, and 1 mosquito per tube. These spiked mosquitoes were extracted using the “one-tube” method and evaluated on the RT-LAMP assay as above. Each reaction was performed with two technical replicates, the RRV RNA synthetic positive control and an NTC.

### 2.6. Assay Validation With Field Samples Using the RRV RT-LAMP Assay and Gold-Standard RT-qPCR

Mosquitoes were collected throughout Maroondah Council, Victoria, as above, following reports of disease activity from November 2023 to March 2024. Pools of 5–10 mosquitoes underwent RNA extraction using the “one-tube” protocol described in the previous section. Each mosquito pool was quantified using the Qubit RNA High Sensitivity Kit (Thermo Fisher Scientific Pty Ltd) to verify successful extraction. Five microlitres of each crude sample were tested in duplicates using the RRV RT-LAMP assay, with a fluorescence threshold ration of 0.01 normalised fluorescence units. Each assay additionally included the RRV synthetic positive control. Results were evaluated as above, where samples were considered positive for RRV if each replicate amplified prior to 20 min.

The homogenised mosquitoes were then subjected to further purification for evaluation using the gold-standard RT-qPCR [41]. Extraction and purification were performed utilising a previously published extraction protocol [42], with minor amendments. Briefly, 100 µl of the macerated mosquitoes was combined with 10 µl of Proteinase K (20 mg/µl) and incubated for 1 h at 55°C. After centrifugation for 10 min at 13,000× *g*, the supernatant was mixed with an equal volume of isopropanol and incubated at −20°C for 45 min. After incubation, the samples were centrifuged for 5 min at 13,000× *g* and pellets were washed with 70% (*v*/*v*) ethanol followed by a further centrifugation for 2 min at 13,000× *g*. The aqueous phase was then removed, and tubes were air dried at room temperature for 15 min. The dried pellets were resuspended in 40 µl of dH_2_O and stored at −20°C until use. Five microlitres of purified mosquito samples were evaluated on RT-qPCR using previously published primers (0.8 µM) [41], with 10 µl of Luna Universal One-Step reaction mix (New England Biolabs, Massachusetts, United States), 1 µl of Luna WarmStart reverse transcription enzyme, and brought to 20 µl with dH_2_O. Reactions were performed on a magnetic induction cycler (MIC) qPCR instrument (Bio Molecular Systems, Queensland, Australia) under the following conditions: 55°C for 10 min, followed by 40 cycles of 95°C for 15 s and 57°C for 45 s. Each sample was performed in duplicates. Data analysis was performed on micPCR software (V2.12.7) using a LinRegPCR method and reported as quantification cycle (Cq) values with a fluorescence cutoff of 0.05%. A sample was considered positive if the Cq value was <40 for both duplicate reactions.

For both the RT-qPCR and RRV RT-LAMP assay, a sample was considered “inconclusive” if only one replicate returned a positive result. The clinical sensitivity of the RRV RT-LAMP assay was determined by assessing its ability to correctly identify true positive and true negative samples. Correct identification was defined as yielding the same result as RT-qPCR, specifically, returning either 2/2 positive or 2/2 negative results. Results that returned inconclusive were repeated where possible.

## 3. Results

### 3.1. Optimisation of the RRV RT-LAMP Assay

Initial assessment and optimisation of the RRV RT-LAMP primers were performed using 1 ng/µl (6.24 × 10^9^ copies/µl) of the synthetic positive control as template in each reaction with various primer concentrations. Each primer concentration combination returned a positive result within 20 min (Table 2), and therefore the optimal concentration was selected based on the lowest yielding Tp and inter-CV. It was determined a final concentration of 0.2 µM for F3 and B3, 1.6 µM for FIP and BIP, and 1.0 µM for LB (Combination 3) were optimal (Table 2). Concentrations either higher or lower for FIP, BIP, and LB resulted in a delay of the average Tp.

Using primer Combination 3, various temperatures were evaluated, and it was revealed the optimal amplification temperature to be 64°C (Table 3).

### 3.2. Analytical Performance and Optimisation With TMAC

**Figure 2:**
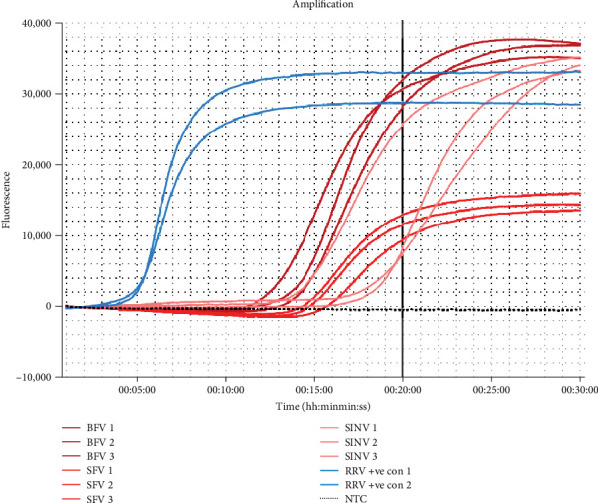
Amplification curve provided by the Genie II machine (OptiGene Limited) for the specificity panel used to determine the RRV reverse-transcription loop-mediated isothermal amplification (RRV RT-LAMP) assay, using synthetic controls of closely related pathogens (Barmah Forest virus—BFV, dark red; Semliki Forest virus—SFV, red; Sindbis virus—SINV, light red). Each specificity sample was performed in triplicate technical replicates. The RRV RNA synthetic positive control (RRV +ve con) is denoted in blue, and the no template control (NTC) is represented in black. Each specificity sample showed cross-reactivity with the RRV RT-LAMP assay, returning a positive result within the 20-min threshold (represented as a black solid vertical line). RRV, Ross River virus.

During testing of closely related viruses BFV, SFV, and SINV, our assay showed low discrimination between pathogens, with amplification occurring within 20 min for each specificity target, although inconsistently. The cross-reactivity resulted in the RRV RT-LAMP to have a low specificity of 55% (Figure 2).

**Figure 3:**
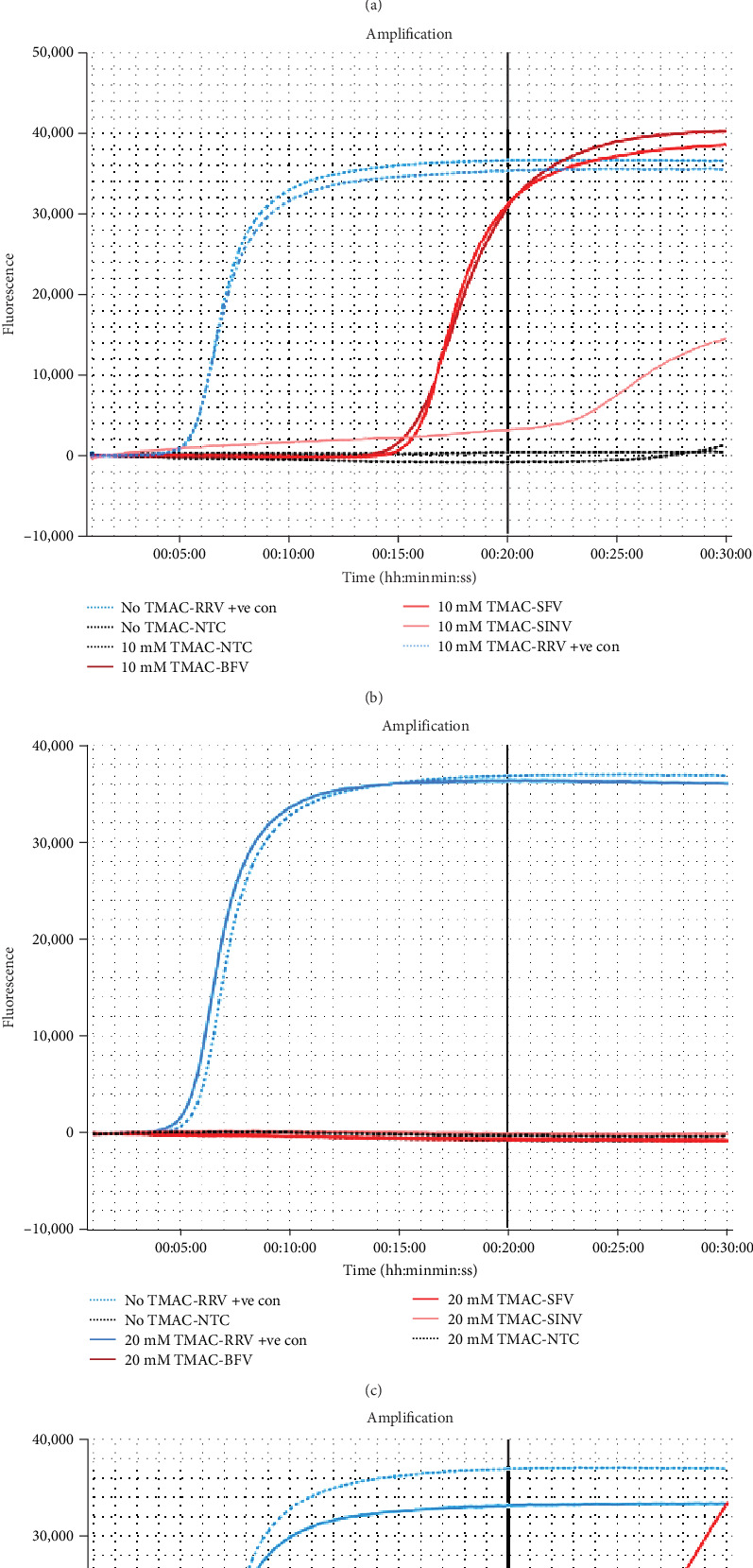
Performance of various tetramethylammonium chloride (TMAC) concentrations evaluated for the suppression of nontarget amplification in the RRV reverse-transcription loop-mediated isothermal amplification (RRV RT-LAMP) assay. Each assay contained the RRV RNA synthetic positive control (RRV +ve con) without the addition of TMAC (blue dotted line) and with the testing concentration of TMAC (blue solid line), as well as no template controls (NTC) with and without TMAC (black dotted lines). Amplification curve plots provided by the Genie II machine (OptiGene) for TMAC concentrations of (a) 5 mM, (b) 10 mM, (c) 20 mM, and (d) 40 mM. RRV, Ross River virus.

To combat the cross-reactivity, the use of TMAC was evaluated to supress non-target amplification at final concentrations of 5, 10, 20, and 40 mM (Figure 3). There was no suppression of non-target amplification using 5 mM of TMAC, with all closely related viruses amplifying within 20 min (Figure 3a). Likewise, using 10 mM of TMAC only suppressed amplification of SINV, with both BFV and SFV amplifying at ~17 min (Figure 3b). In contrast, both 20 and 40 mM were able to suppress amplification of each closely related pathogen beyond the 20-min threshold (Figure 3c,d). Interestingly, all concentrations of TMAC demonstrated no Tp delay of the RRV synthetic positive control (at ~6 min and 30 s). It was determined that 20 mM of TMAC was the optimal concentration due to the most consistency in the suppression of non-target amplification. This increased the RRV RT-LAMP assay's specificity to 100%.

To determine the LOD of the RRV RT-LAMP assay with 20 mM TMAC, serial dilutions of the synthetic positive control were performed (*n* = 3). The LOD was defined as the lowest concentration which a Tp for each replicate amplified within 20 min, resulting in a detection limit of 1 × 10^−7^ ng/µl (~6.24 × 10^2^ copies/µl) with an inter CV range of 0.38–5.62% (Table 4). Lower concentrations of 1 × 10^−8^ ng/µl (~62.4 copies/µl) could be detected within the time threshold at times, the results were not consistent across multiple assays and thus are not being reported as the LOD. The LOD was also calculated for the assay without the addition of TMAC, to observe any assay inhibitions that could arise from using the chemical. Whilst it was noted the RRV RT-LAMP assay without TMAC showed an increase of LOD (~62.4 copies/µl), the use of 20 mM TMAC not only had a far superior specificity but decreased the average Tp by ~3 min (Table 4).

#### 3.2.1. Mosquito Spiking Sample Preparation and RNA Extraction

**Figure 4:**
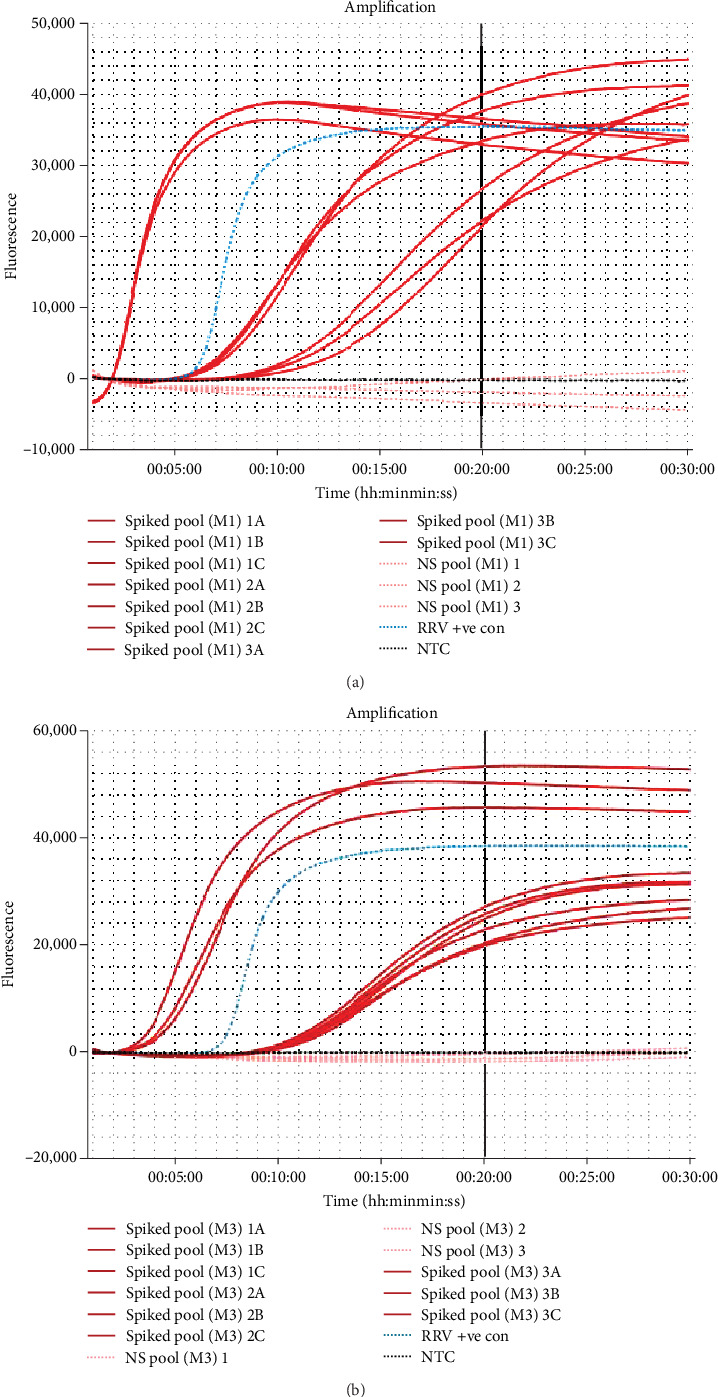
Amplification curve plots provided by Genie II machine (OptiGene Limited) for the RNA extraction from mosquitoes using the RRV reverse-transcription loop-mediated isothermal amplification (RRV RT-LAMP) assay. Both assays contained the RRV RNA synthetic positive control (RRV +ve con, blue), three non-spiked mosquito pools (NS pool—light red) and a no template control (NTC—black). The time to positive (Tp) threshold at 20 min is denoted as a black solid line. (a) Spiked mosquito pools (red) amplification for RNA extraction using the “in-tube” method. (b) Spiked mosquito pools amplification for RNA extraction using the “one-tube” method. RRV, Ross River virus.

Pools of 5–10 mosquitoes spiked with the RRV synthetic positive control were processed using one of the three extraction methods described. Both the “in-tube” and “one-tube” RNA extraction techniques were successfully able to extract RNA from mosquitoes consistently. However, the “in-syringe” method failed to extract RNA from every sample (3/6), as such this method was excluded from further testing and evaluation. The “in-tube” method yielded the highest concentrations of RNA, between 10 and 20 ng/µl; however, the additional use of a centrifuge required for bulk sampling processing makes in-field application difficult. The “one-tube” method had a lower average RNA yield, between 6 and10 ng/µl, but allows for in-field use. Sanger sequencing revealed that both extraction methods were able to successfully extract detectable RRV RNA in the spiked mosquitoes (Table S1). Both the “in-tube” and “one-tube” RNA extraction methods were able to successfully amplify all spiked mosquitoes. Additionally, none of the non-spiked mosquitoes amplified from either method (Figure 4). The “one-tube” extraction method demonstrated the most consistent amplification on the spiked mosquitoes. Based on these results, and the simplicity of extraction, it was decided to continue with the “one-tube” method for the remaining mosquito RNA extractions.

**Figure 5:**
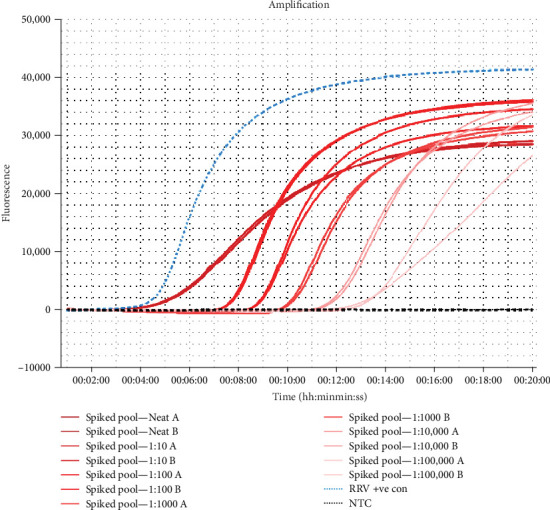
Amplification curve of RRV RT-LAMP assay on serially diluted spiked mosquitoes from neat to 1:100,000, extracted using the “one-tube” method. Each dilution returned a Tp within 20 min. The LOD was determined to be 1:10,000. RRV, Ross River virus.

**Figure 6:**
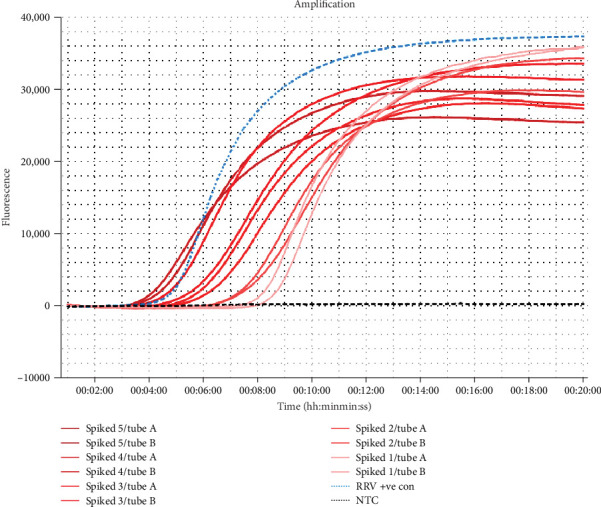
RRV RT-LAMP assay amplification curve using a range of five spiked mosquitoes per tube to one spiked mosquito per tube. Mosquitoes were incubated with the RNA RRV synthetic positive control (0.1 ng/µl final concentration) in bulk (*n* = 5) and aliquoted into respective numbers per tube. All mosquitoes were extracted using the “one-tube” method. Each sample returned a Tp within 20 min. RRV, Ross River virus.

Mosquitoes spiked with serially diluted control RNA were able to consistently amplify a dilution of 1:10,000 using the one-tube method (Figure 5). Although a dilution of 1:100,000 could amplify at times (Figure 5), this was not demonstrated in each repeated assays and therefore was excluded from the calculation. Evaluation of number of spiked mosquitoes per tube revealed the assay could detect RRV in just one spiked mosquito (Figure 6).

### 3.3. Assay Validation on Field Samples and the Gold-Standard RT-qPCR

A total of 102 mosquito pools, containing 5–10 mosquitoes each, were evaluated using the RRV RT-LAMP assay and RT-qPCR assay. The RRV RT-LAMP assay could not determine whether four mosquito samples were positive or negative for RRV. Likewise, the RT-qPCR returned inconclusive results for four samples; however, these were different from the inconclusive RT-LAMP assay results. Each of these eight samples was unable to return definitive results upon re-testing on their respective molecular assays. Consequently, these samples were excluded from further calculations, resulting in a final total of 94 mosquito pool samples. The RRV RT-LAMP assay detected 83 (88%) mosquito pools as positive for RRV and 11 (12%) as negative (Table 5), with a Tp ranging from 01:47 to 19:53.

The RT-qPCR assay identified 82 samples (87%) as positive, and 12 (13%) as negative. Out of the 94 samples analysed, there was consensus of 79 results between the two assays. Amongst these agreed-upon results, 75 (95%) were identified as positive, while 4 (5%) were negative, with 15 (16%) samples remaining in disagreement. Based on the assumption, the RT-qPCR obtains a 100% accuracy, this yields the RRV RT-LAMP assay with a clinical sensitivity of 84% (Table 5) (see Table S2 for full results).

## 4. Discussion

This project successfully developed a rapid RT-LAMP assay to detect the RRV E2 gene in crude mosquito samples. The increasing RRV cases throughout Australia, particularly the spread to inland regions, highlights the importance of continuous and accurate surveillance [13]. However, current surveillance techniques in Australia, such as predictive modelling and RT-qPCR, are not viable for all regions [15, 28]. The use of expensive machinery that require operators to be specially trained leaves many remote and resource-poor communities vulnerable [1]. While predictive modelling was previously useful, the impact of climate change and subsequent extreme weather patterns has rendered this technique cumbersome [16]. Herein, we have generated an in-field applicable mosquito extract technique paired with a specific and sensitive RRV RT-LAMP assay for effective diagnostics.

We aimed to develop our RRV RT-LAMP assay to have a high discrimination against closely related alphaviruses, such as BFV, SFV, and SINV. Previous studies have revealed a single amino acid mutation within the RRV E2 gene at the 218 residues, allowing for differentiation between other alphaviruses [43]. Furthermore, currently circulating strains of RRV, MIDI13.2016, MIDI32.2017, and MIDI86,1028, have shown this area of mutation in the E2 has been highly conserved since 1979 [44, 45]. As such the RRV RT-LAMP primers were designed to encompass this area, where homology between the closely related alphaviruses BFV, SFV, and SINV showed a homology of <50% (Figure S1). Whilst much of literature states LAMP is superior in specificity due to the use of multiple primers targeting different regions of the same gene [46, 47] non-specific and non-target amplification commonly occurs [48–50]. When evaluating the specificity of the RRV RT-LAMP assay against synthetic constructs of BFV, SFV, and SINV, amplification was occurring prior to the 20-min threshold, despite little homology to RRV being less than 50%. As the sequences for the closely related viruses were relatively high (>50%) in AT content, we theorised there was base pair mismatching occurring during cDNA synthesis and/or primer annealing. As base pair stability is partially dependent on hydrogen bonds, it is well recognised that AT base pairs, having only a double hydrogen bond, are less stable than GC base pairs, which have a triple bond [51, 52]. Subsequently, AT base pairs have a lower melting temperature compared to GC base pairs and thus are more prone to base pair mismatching [53]. As such, we aimed to use a chemical additive to strengthen correct AT base pairing.

Previous literature has shown TMAC as a viable option to inhibit AT base pair mismatching and therefore non-target amplification [52]. TMAC preferentially interacts with AT base pairs through its non-polar arm that contains hydrophobic components which interact with the AT base pairs hydrophobic surfaces [49, 52]. This interaction between TMAC and AT base pairs results in a more stable duplex with a higher melting temperature closer to that of GC base pairs, thus reducing base pair mismatching during amplification procedures [52, 54]. Jang and Kim [49] demonstrated the use of TMAC in RT-LAMP using a range of final concentrations of 20–100 mM, increasing by twofold. The paper found a concentration of 20 mM was able to suppress non-specific amplification whilst limiting the adverse effects on the assay itself. The authors noted concentrations of 80 and 100 mM increase the RT-LAMP's SD by four- and half-fold. Our findings agreed with Jang and Kim [49], where the optimal final concentration of TMAC in our assay was 20 mM, which suppressed non-target amplification of BFV, SFV, and SINV.

We evaluated the RRV RT-LAMP assay's LOD with and without the addition of TMAC, to observe any suppression in assay kinetics. With the use of 20 mM TMAC, the calculated LOD was 1 × 10^−7^ ng/µl (~624 copies/µl), within roughly 15 min and 30 s.

However, whilst Jang and Kim [49] observed no effect on other optimisation steps for their RT-LAMP assay [49], the LOD for the RRV RT-LAMP without the use of TMAC was 10-fold higher at 1 × 10^−8^ ng/µl (~62.4 copies/µl), on par with the LOD reported for the gold-standard RT-qPCR assay. However, the Tp for 1 × 10^−7^ ng/µl without the addition of TMAC was roughly 3 min slower. Despite the reduction of analytical sensitivity associated with TMAC, the benefit of a highly specific assay is far more desirable. Additionally, the decrease in Tp when using TMAC renders our assay to be over five times faster than the RT-qPCR assay. Therefore, the use of TMAC in RT-LAMP assays could greatly benefit future surveillance techniques for the discrimination of closely related pathogens [43].

In our study, we assessed three field-deployable RNA extraction techniques, adapted from previously published literature. Of the three methods, the “in-tube” extraction yielded the highest average concentration of RNA in spiked mosquitoes. However, as the original protocol was optimised to extract from just a single mosquito [39], we decided to include a table-top microcentrifuge to separate remaining mosquito debris from the supernatant, with the intention to extract bulk samples. Whilst field-deployable battery-operated centrifuges are available, this would inevitably increase the cost of sample processing [55]. As such, two other RNA extraction methods without the use of machinery were examined. The “in-syringe” method developed by Bhadra et al. [40] was a promising alternative for sample extraction that minimised the use of disposable products whilst effectively eliminating crushed mosquito debris from the tube. However, inserting the 0.5 µM pore frit into the 1 cc syringe presented challenges, as manual application of the frit into the syringe nozzle was occasionally unsuccessful, thus leading to inconsistent maceration of the mosquito samples. As a result, RNA extraction using the “in-syringe” was not evaluated for each extraction. Subsequently, we developed an RNA extraction that combined features from both previous methods. This “one-tube” technique integrates the simplicity of homogenising mosquito tissue with a pestle as per Lamb et al. [39] and employs a syringe filter to remove mosquito debris, akin to the approach described by Bhadra et al. [40]. Moreover, the application of mechanical lysis, as outlined by Bhadra et al. [40], has enabled our “one-tube” RNA extraction method to operate without the need for specialised lysis buffers, providing a more cost-effective sample preparation alternative. Our developed method was able to consistently extract RNA and detect RRV in the spiked mosquitoes in the RRV RT-LAMP assay.

The “one-tube” sample extraction procedure can be performed in under 2 min, whereas samples had to undergo further purification steps to remove inhibitors from the samples for the RT-qPCR assay. It is hypothesised the *Bst* polymerase RT-LAMP employs greatly tolerates sample inhibitors without compromising on assay quality or performance [33], a sentiment that has been acknowledged throughout numerous studies [40, 56, 57]. Coupled with the reverse-transcriptase enzyme, RT-LAMP's ability to be performed on minimally processed or crude samples without the need for separate RT procedures, further establishes its use for in-field testing. Allowing for extraction, RT, and amplification to be completed in just two steps greatly aids in timely interventions and prevention of disease transmission [29].

During testing of field-caught mosquitoes, eight pools returned “inconclusive” results. Whilst both assays had a final of four inconclusive results each, it should be noted the RT-qPCR originally had 20 inconclusive results before retesting. The RRV RT-LAMP assay determined 83 mosquito pools were positive for RRV, in which 75 (90%) of those were detected on the RT-qPCR. Yet only 4 of the 11 (36%) samples that returned a negative result by RT-LAMP also indicated negative on RT-qPCR, which may be due to the latter being more analytically sensitive, or the RRV RT-LAMP produced false-negative results. However, the RRV RT-LAMP assay detected 8 of 12 (66%) RT-qPCR negative samples as positive. While this may suggest the RRV RT-LAMP yielded false-positive results, it is also possible inhibitors remained in these samples after purification, which prevented amplification by the RT-qPCR assay. This solidifies the unique advantage LAMP-based technology has over impurity tolerance over other nucleic acid amplification procedures. Despite this disparity, we opted to presume the RT-qPCR has 100% accuracy. As a result, we calculated the RRV RT-LAMP clinical sensitivity to be 84% (79/94 correct identifications). However, it is worth noting the clinical sensitivity of the RT-qPCR assay has not been reported [13, 41].

Although the RRV RT-LAMP assay has a marginally lower LOD than the RT-qPCR, this assay can be utilised as a rapid routine mosquito surveillance procedure which can provide results in under 30 min from sample collection. However, to facilitate this assay in remote areas, it is recommended that the sample processing technique is optimised to upscale the number of mosquitoes per tube. While surveying ~10 mosquitoes at a time allows for a more specific and targeted result within a region, bulk sample processing may be more practically feasible. Furthermore, RT-LAMP optimisation into a result output which can be viewed by the naked eye, such as a colorimetric assay, may be beneficial for resource-poor settings and to reduce costs. Nevertheless, the RRV RT-LAMP assay provides a promising alternative to mosquito surveillance programs throughout Australia.

## 5. Conclusions

We have developed and optimised a reliable and robust RT-LAMP assay for the detection of RRV in mosquitoes. Coupled with the straightforward RNA extraction technique determined in this investigation, this assay is designed for deployment in field settings. The assay is capable of consistently detecting synthetic RRV RNA in just a single mosquito within 20 min post-sample collection. Our assay was able to detect RRV in field samples, echoing similar results to the gold-standard RT-qPCR assay. Incorporating TMAC effectively mitigated non-target amplification of closely related alphaviruses, making our assay highly specific. Our RRV RT-LAMP assay offers a cost-effective and timely alternative to existing surveillance techniques, requiring minimal equipment and thereby facilitating its utilisation in resource-limited and rural communities. The prevalence of RRV is continually expanding in Australia with reliable epidemiology techniques paramount to ensuring large outbreaks are mitigated before their occurrence.

## Figures and Tables

**Table 1:** Primers designed for the Ross River virus reverse-transcription loop-mediated isothermal amplification (RRV RT-LAMP) assay targeting the envelop protein 2 gene (E2) (accession no: KX709870.1).

Primer	Length (bp)	Genome position (nt)	Sequence (5′-3′)
RRVE2_F3	20	8931–8950	GCGACTACCTCAAGGTTTCA

RRVE2_B3	19	9124–9142	CGGTCTATATGGCCTAGCG

RRVE2_FIP	40	8960–8978 (F1c) 9000–9020 (F2)	TACCCACCGGCAATGGATCGT GCAGATTCGCACGTGAAGG

RRVE2_BIP	42	9098–9117 (B1c) 9039–9060 (B2)	GACCACACTTTGGCGTAGAGCT TGCATGTCAATCTCCTCGTC

RRVE2_LB	23	9066–9088	GTACCTCATACCAGCTGACAACG

*Note:* Genome position is based on accession no: MH987780.1. Sequences underlined represent reverse complement.

**Table 2:** Assessed primer concentration and results for the RRV RT-LAMP assay primer set utilised in this study.

Primer combination	Primer concentration (F3/B3; FIP/BIP; LB)	Average Tp (minmin:ss)	Interassay CV (%)
Combination 1	0.2 µM; 0.8 µM; 0.4 µM	09 : 01 ± 0 : 07 SD	0.19
Combination 2	0.2 µM; 1.6 µM; 0.8 µM	07 : 57 ± 0 : 07 SD	0.17
Combination 3	0.2 µM; 1.6 µM; 1.0 µM	06 : 41 ± 0 : 01 SD	0.03

Abbreviations: CV, coefficient of variation; Tp, time to positive.

**Table 3:** Assessed amplification temperatures and results for the RRV RT-LAMP assay.

Amplification temperature (°C)	Average Tp (minmin:ss)	Inter-assay CV (%)
62	06:19 ± 0:07	0.13
63	07:01 ± 0:31	1.00
64	05:58 ± 0:09	0.16

Abbreviations: CV, coefficient of variation; Tp, time to positive.

**Table 4:** RRV RT-LAMP limit of detection and inter–coefficient of variation (CV) comparison with the use of tetramethylammonium chloride (TMAC).

Starting template concentration (ng/µl)	0 mM TMAC		20 mM TMAC		
	Average Tp^a^ (minmin:ss)	Inter-CV (%)		Average Tp^b^ (minmin:ss)	Inter-CV (%)
1 × 10^−1^	07:43 ± 0:33 SD	0.74		05:38 ± 0:03 SD	0.38
1 × 10^−2^	09:42 ± 0:44 SD	1.25		07:06 ± 0:31 SD	0.64
1 × 10^−3^	12:21 ± 1:36 SD	3.44		08:24 ± 0:41 SD	1.00
1 × 10^−4^	13:31 ± 0:53 SD	2.10		10:42 ± 1:10 SD	2.19
1 × 10^−5^	15:31 ± 1:17 SD	3.48		11:30 ± 0:36 SD	1.23
1 × 10^−6^	16:02 ± 0:34 SD	1.60		14:41 ± 2:09 SD	5.52
1 × 10^−7^	18:27 ± 1:06 SD	3.57		15:32 ± 2:05 SD	5.62
1 × 10^−8^	19:47 ± 0:36 SD	2.25		—	—
1 × 10^−9^	—	—		—	—

Abbreviations: SD, standard deviation; Tp, time to positive.

^a^Average Tp of three technical replicates in three assays (*n* = 9 replicates per concentration).

^b^Average Tp of three technical replicates in five assays (*n* = 15 replicates per concentration).

**Table 5:** Comparison of identified mosquito pool samples between the RRV RT-LAMP assay and RT-qPCR assay.

Diagnostic test	Number of positive	Number of negative	Total
RRV RT-LAMP	83	11	94
RT-qPCR	82	12	94

*Note:* Agreement and disagreement are calculated based on the assumption that the RT-qPCR has correctly identified all results.

## Data Availability

All data are contained within this article or in the supporting information.

## Supporting Information

Supporting Information Figure S1. sequence pairwise alignment of closely related alphaviruses Barmah Forest virus (BFV—S1a), Semliki Forest virus (SFV—S1b), and Sindbis virus (SINV—1c) E2 gene, against the Ross River virus (RRV) E2 gene. RRV reverse-transcription loop-mediated isothermal amplification (RT-LAMP) primers are highlighted throughout, outer primers (F3 and B3) are labelled in yellow, forward inner primers (F2 and F1c) are highlighted in blue, the backwards inner primers (B2 and B1c) are highlighted in green, and the backwards loop primer (LoopB) is highlighted in grey. Sequence consensuses are highlighted in red. Alignment was performed using Benchling online software (Benchling, San Fransico, United States) and edited in Microsoft Word (Microsoft Corporation, Washington, United States). Table S1. Sanger sequencing result example provided by AGRF (Melbourne, Victoria) from a mosquito pool spiked with the RRV synthetic positive control. Table S2. raw data results from the RRV reverse-transcription loop-mediated isothermal (RRV RT-LAMP) assay and the reverse-transcription quantitative polymerase chain reaction (RT-qPCR) assay on collected mosquitoes.
