# Supplementary material for: Development of a Rapid Surveillance System for Ross River Virus in Mosquitoes Through Reverse-Transcription Loop-Mediated Isothermal Amplification (RT-LAMP)
Source: Transbound Emerg Dis. 2025 Feb 28;2025:1772438. doi: 10.1155/tbed/1772438 (PMC12017211; doi:10.1155/tbed/1772438)
Supplement: Supporting Information — Figure S1. sequence pairwise alignment of closely related alphaviruses Barmah Forest virus (BFV—S1a), Semliki Forest virus (SFV—S1b), and Sindbis virus (SINV—1c) E2 gene, against the Ross River virus (RRV) E2 gene. RRV reverse-transcription loop-mediated isothermal amplification (RT-LAMP) primers are highlighted throughout, outer primers (F3 and B3) are labelled in yellow, forward inner primers (F2 and F1c) are highlighted in blue, the backwards inner primers (B2 and B1c) are highlighted in green, and the backwards loop primer (LoopB) is highlighted in grey. Sequence consensuses are highlighted in red. Alignment was performed using Benchling online software (Benchling, San Fransico, United States) and edited in Microsoft Word (Microsoft Corporation, Washington, United States). Table S1. Sanger sequencing result example provided by AGRF (Melbourne, Victoria) from a mosquito pool spiked with the RRV synthetic positive control. Table S2. raw data results from the RRV reverse-transcription loop-mediated isothermal (RRV RT-LAMP) assay and the reverse-transcription quantitative polymerase chain reaction (RT-qPCR) assay on collected mosquitoes. [file 1772438.f1.docx]

**“Development of a rapid surveillance system for the detection of Ross River virus (RRV) in mosquitoes through reverse-transcription loop-mediated isothermal amplification (RT-LAMP)**

Alexandra Knox and Travis Beddoe

**Supplementary materials**

**
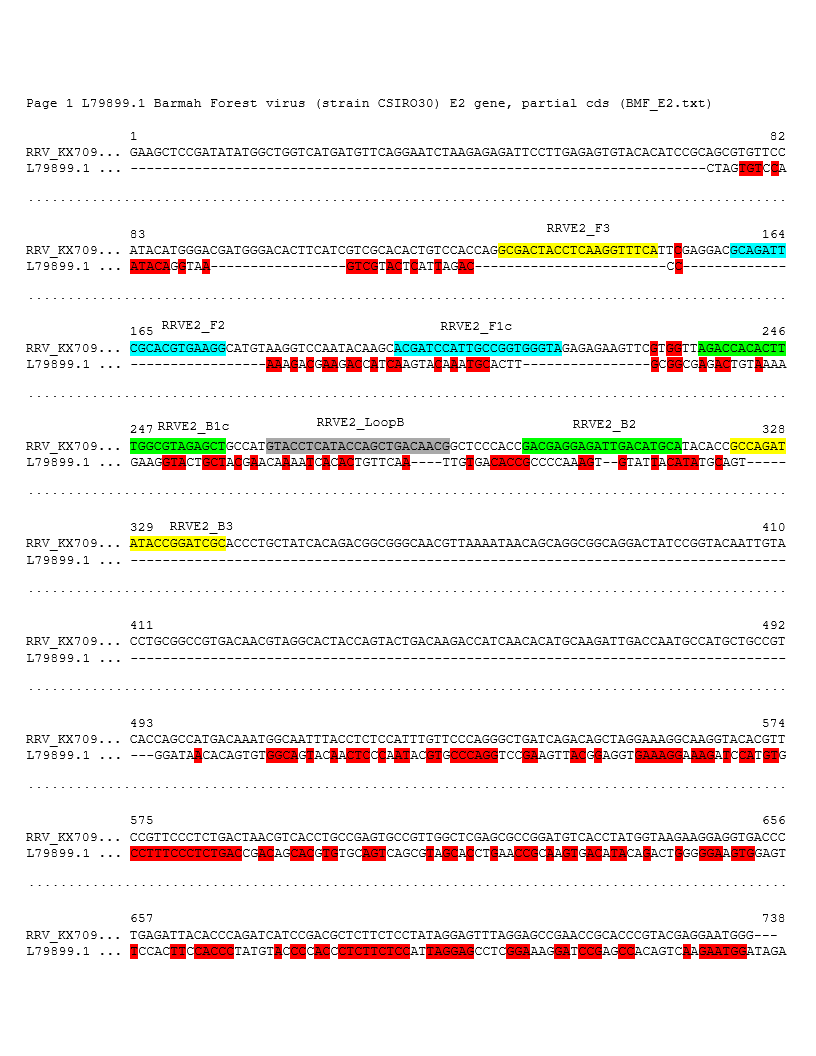
**

**
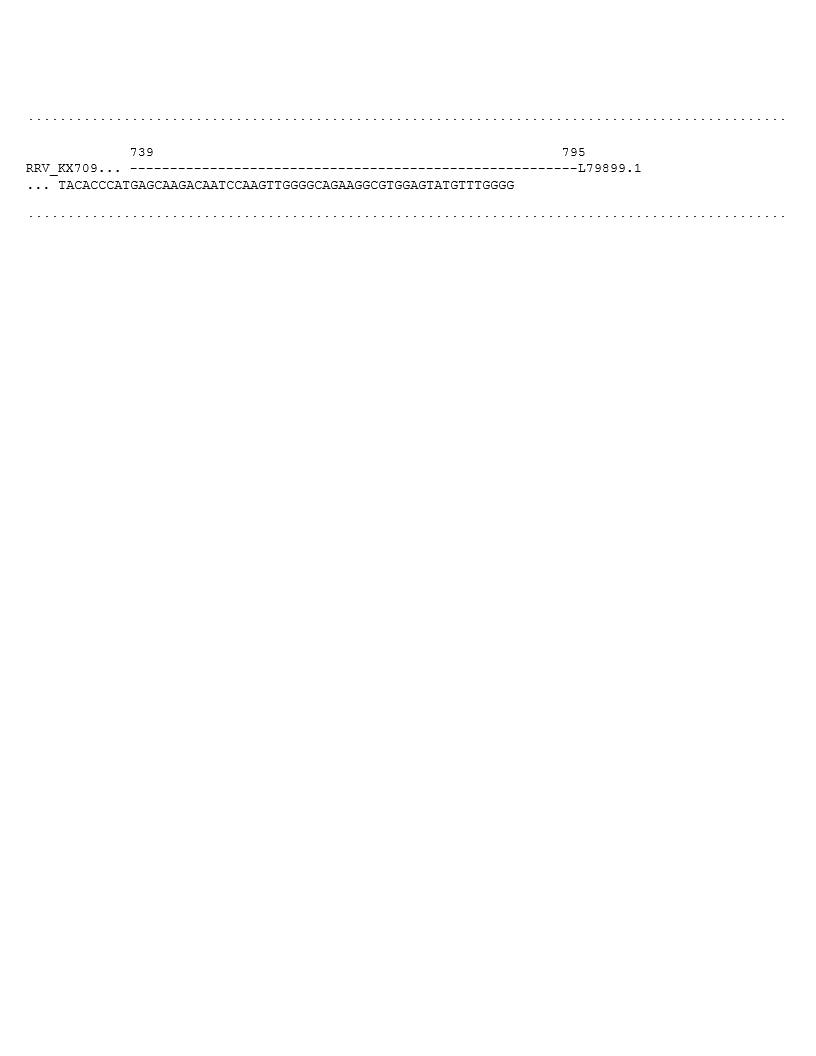
**

**a**

**
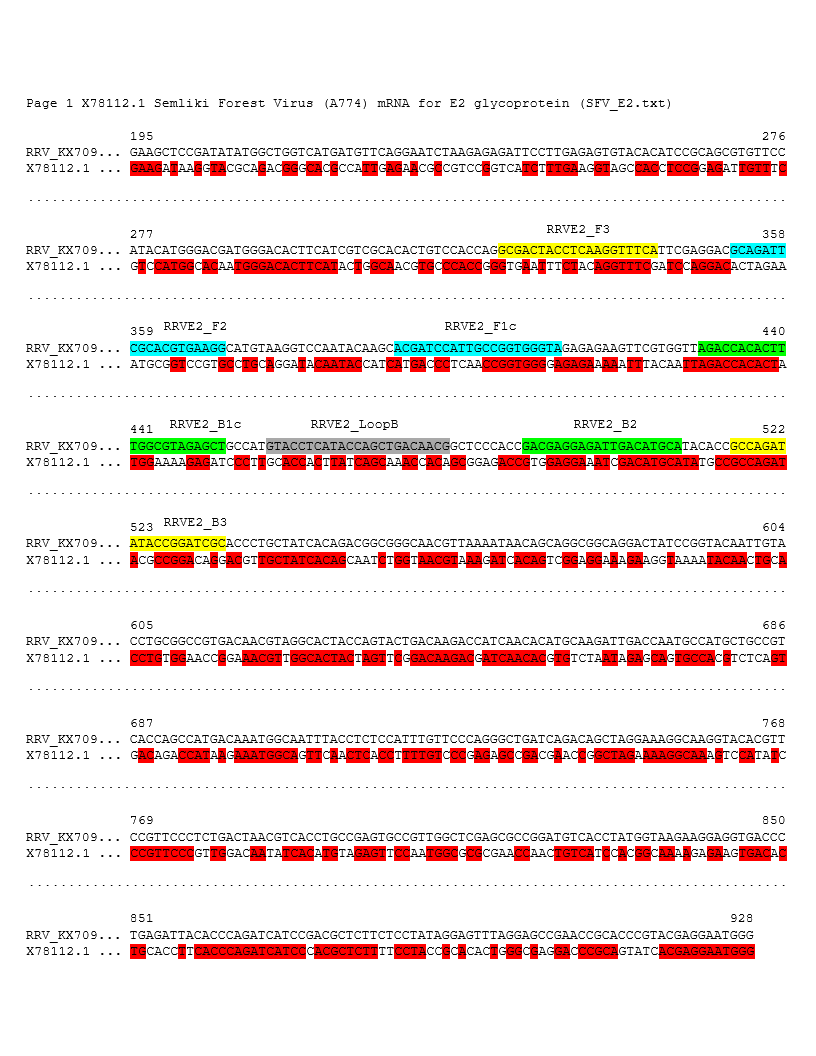
**

**b**

**
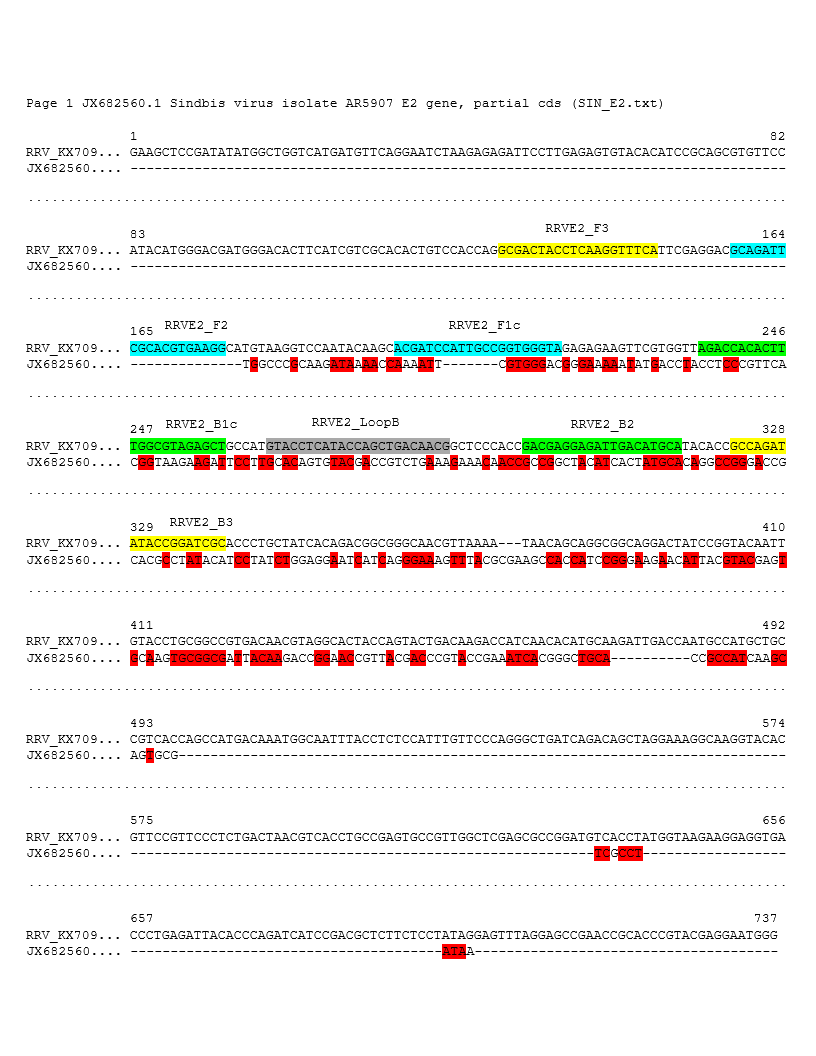
**

**c**

Figure S1: Sequence pairwise alignment of closely related alphaviruses Barmah Forest virus (BFV – S1a), Semliki Forest virus (SFV – S1b), and Sindbis virus (SINV – 1c) E2 gene, against the RRV E2 gene. RRV RT-LAMP primers are highlighted throughout, outer primers (F3 and B3) are labelled in yellow, the forward inner primers (F2 and F1c) are highlighted in blue, the backwards inner primers (B2 and B1c) are highlighted in green, and the backwards loop primer (LoopB) is highlighted in grey. Sequence consensuses are highlighted in red. Alignment was performed using Benchling online software (Benchling, San Fransico, United States), and edited in Microsoft Word (Microsoft Corporation, Washington, United States).

**b**

Table S1: Sanger Sequencing result example provided by AGRF (Melbourne, Victoria) from a mosquito pool spiked with the Ross River virus (RRV) synthetic positive control.

| Target/Primer | Sequence obtained (5’-3’) | BLAST result |
| --- | --- | --- |
| RRV E2 gene  Forward primer | TCGGGAATCCCGTGAAGGCATGTAAGGTCCAATACAAGCACGATCCATTGCCGGTGGGTAGAGAGAAGTTCGTGGTTAGACCACACTTTGGCGTAGAGCTGCCATGTACCTCATACCAGCTGACAACGGCTCCCACCGACGAGGAGATTGACATGCATACACCGCCAGATATACCGGATCGCAAAC | KY290876.1 Ross River virus isolate 19776 structural polyprotein |
| RRV E2 gene  Reverse primer | NAAACAAACTTTCNGAGGTTAATCTCCTCGTCGGTGGGAGCCGTTGTCAGCTGGTATGAGGTACATGGCAGCTCTACGCCAAAGTGTGGTCTAACCACGAACTTCTCTCTACCCACCGGCAATGGATCGTGCTTGTATTGGACCTTACATGCCTTCACGTGCGAATCTGCGTCCTCGAATGAAACCTTGAGGTAGTCGCAA | KY290876.1 Ross River virus isolate 19776 structural polyprotein |

Table S2: Raw data results from the Ross River virus reverse-transcription loop-mediated isothermal (RRV RT-LAMP) assay and the reverse-transcription quantitative polymerase chain reaction (RT-qPCR) assay on collected mosquitoes.

| **Sample** | **RT-LAMP result** | **Tp (mm:ss)** | **RT-LAMP decision** | **RT-qPCR Result** | **Cq** | **RT-qPCR decision** | **Agreement?** |
| --- | --- | --- | --- | --- | --- | --- | --- |
| S01 A | POS | 7:44 | POS | POS | 33.26 | POS | Y |
| S01 B | POS | 7:43 |  | POS | 32.66 |  |  |
| S02 A | POS | 5:24 | POS | POS | 32.66 | POS | Y |
| S02 B | POS | 5:26 |  | POS | 31.91 |  |  |
| S03 A | POS | 3:34 | POS | POS | 32.23 | POS | Y |
| S03 B | POS | 3:38 |  | POS | 3.9 |  |  |
| S04 A | NEG | - | NEG | POS | 32.48 | POS | N |
| S04 B | NEG | - |  | POS | 17.23 |  |  |
| S05 A | POS | 13:50 | POS | POS | 34.22 | POS | Y |
| S05 B | POS | 14:13 |  | POS | 32.39 |  |  |
| S06 A | POS | 11:14 | POS | POS | 30.43 | POS | Y |
| S06 B | POS | 12:13 |  | POS | 32.34 |  |  |
| S07 A | POS | 6:44 | POS | POS | 33.1 | POS | Y |
| S07 B | POS | 6:36 |  | POS | 32.05 |  |  |
| S08 A | POS | 8:00 | POS | POS | 35.5 | POS | Y |
| S08 B | POS | 7:57 |  | POS | 31.63 |  |  |
| S09 A | POS | 3:05 | POS | POS | 31.78 | POS | Y |
| S09 B | POS | 3:03 |  | POS | - |  |  |
| S10 A | POS | 2:49 | POS | POS | 36.42 | POS | Y |
| S10 B | POS | 2:51 |  | POS | 32.89 |  |  |
| S11 A | POS | 4:50 | POS | NEG | - | INC | P INC |
| S11 B | POS | 4:55 |  | POS | 31.36 |  |  |
| S12 A | POS | 7:44 | POS | POS | 32.26 | POS | Y |
| S12 B | POS | 7:45 |  | POS | 31.78 |  |  |
| S13 A | POS | 7:29 | POS | POS | 33.01 | POS | Y |
| S13 B | POS | 6:29 |  | POS | 32.64 |  |  |
| S14 A | POS | 7:33 | POS | POS | 31.61 | POS | Y |
| S14 B | POS | 7:35 |  | POS | 34.17 |  |  |
| S15 A | POS | 3:27 | POS | POS | 34.3 | POS | Y |
| S15 B | POS | 3:22 |  | POS | 34.85 |  |  |
| S16 A | POS | 2:55 | POS | POS | 28.33 | POS | Y |
| S16 B | POS | 2:56 |  | POS | 31.55 |  |  |
| S17 A | POS | 5:10 | POS | POS | 31.15 | POS | Y |
| S17 B | POS | 5:35 |  | POS | 31.48 |  |  |
| S18 A | POS | 8:04 | POS | POS | 34.33 | POS | Y |
| S18 B | POS | 7:58 |  | POS | 32.67 |  |  |
| S19 A | POS | 6:18 | POS | POS | 32.31 | POS | Y |
| S19 B | POS | 6:03 |  | POS | 36.47 |  |  |
| S20 A | POS | 7:28 | POS | POS | 33.09 | POS | Y |
| S20 B | POS | 7:52 |  | POS | 32.66 |  |  |
| S21 A | POS | 2:46 | POS | POS | 34.82 | POS | Y |
| S21 B | POS | 2:48 |  | POS | 32.47 |  |  |
| S22 A | POS | 2:34 | POS | POS | 3.01 | POS | Y |
| S22 B | POS | 2:47 |  | POS | 33.29 |  |  |
| S23 A | POS | 5:22 | POS | POS | 33.66 | POS | Y |
| S23 B | POS | 5:43 |  | POS | 31.8 |  |  |
| S24A | POS | 7:10 | POS | POS | 35.45 | POS | Y |
| S24 B | POS | 7:14 |  | POS | 33.32 |  |  |
| S25 A | POS | 2:36 | POS | POS | 34.13 | POS | Y |
| S25 B | POS | 2:36 |  | POS | 33.11 |  |  |
| S26 A | POS | 5:17 | POS | NEG | - | NEG | N |
| S26 B | POS | 5:30 |  | NEG | - |  |  |
| S27 A | NEG | - | NEG | POS | 35.96 | POS | N |
| S27 B | NEG | - |  | POS | 32.32 |  |  |
| S28 A | POS | 6:41 | POS | POS | 32.86 | POS | Y |
| S28 B | POS | 6:49 |  | POS | 32.69 |  |  |
| S29 A | POS | 8:29 | POS | POS | 30.1 | POS | Y |
| S29 B | POS | 8:18 |  | POS | 29.86 |  |  |
| S30 A | POS | 2:49 | POS | POS | 31.92 | POS | Y |
| S30 B | POS | 3:06 |  | POS | 36.52 |  |  |
| S31 A | POS | 5:34 | POS | POS | 30.22 | POS | Y |
| S31 B | POS | 5:44 |  | POS | 31.73 |  |  |
| S32 A | POS | 8:17 | POS | POS | 31.91 | POS | Y |
| S32 B | POS | 7:55 |  | POS | 33.08 |  |  |
| S33 A | POS | 2:39 | POS | POS | 31.33 | POS | Y |
| S33 B | POS | 2:52 |  | POS | 32.78 |  |  |
| S34 A | POS | 3:04 | POS | POS | 31.74 | POS | Y |
| S34 B | POS | 3:17 |  | POS | 33.29 |  |  |
| S35 A | POS | 5:01 | POS | POS | 36.33 | POS | Y |
| S35 B | POS | 5:19 |  | POS | 30.93 |  |  |
| S36 A | POS | 7:22 | POS | POS | 33.76 | POS | Y |
| S36 B | POS | 7:45 |  | POS | 32.58 |  |  |
| S37 A | POS | 3:18 | POS | POS | 32.24 | POS | Y |
| S37 B | POS | 3:21 |  | POS | 31.95 |  |  |
| S38 A | POS | 11:13 | POS | POS | 30.2 | POS | Y |
| S38 B | POS | 11:14 |  | POS | 30.75 |  |  |
| S39 A | POS | 4:25 | POS | POS | 32.71 | POS | Y |
| S39 B | POS | 4:22 |  | POS | 31.94 |  |  |
| S40 A | POS | 9:10 | POS | POS | 31.97 | POS | Y |
| S40 B | POS | 9:18 |  | POS | 32.15 |  |  |
| S41 A | POS | 12:33 | POS | POS | 32.03 | POS | Y |
| S41 B | POS | 12:43 |  | POS | 32.51 |  |  |
| S42 A | POS | 2:04 | POS | POS | 31.65 | POS | Y |
| S42 B | POS | 2:04 |  | POS | 32.6 |  |  |
| S43 A | POS | 3:08 | POS | POS | 32.73 | POS | Y |
| S43 B | POS | 3:24 |  | POS | 33.06 |  |  |
| S44 A | POS | 12:45 | POS | POS | 33.25 | POS | Y |
| S44 B | POS | 12:25 |  | POS | 34.28 |  |  |
| S45 A | POS | 4:09 | POS | POS | 31.04 | POS | Y |
| S45 B | POS | 4:21 |  | POS | 32.2 |  |  |
| S46 A | POS | 9:42 | POS | POS | 33.3 | POS | Y |
| S46 B | POS | 9:23 |  | POS | 32.44 |  |  |
| S47 A | POS | 12:05 | POS | NEG | - | NEG | N |
| S47 B | POS | 12:29 |  | NEG | - |  |  |
| S48 A | POS | 2:04 | POS | POS | 32.11 | POS | Y |
| S48 B | POS | 2:03 |  | POS | 30.97 |  |  |
| S49 A | POS | 6:43 | POS | POS | 30.06 | POS | Y |
| S49 B | POS | 6:35 |  | POS | 35.12 |  |  |
| S50 A | POS | 11:40 | POS | POS | 35.37 | POS | Y |
| S50 B | POS | 7:44 |  | POS | 32.31 |  |  |
| S51 A | POS | 2:55 | POS | POS | 32.78 | POS | Y |
| S51 B | POS | 3:07 |  | POS | 31.81 |  |  |
| S52 A | POS | 2:52 | POS | POS | 32.81 | POS | Y |
| S52 B | POS | 2:50 |  | POS | 34.01 |  |  |
| S53 A | POS | 12:22 | POS | POS | 25.39 | POS | Y |
| S53 B | POS | 12:26 |  | POS | 25.39 |  |  |
| S54 A | POS | 4:07 | POS | POS | 30.62 | POS | Y |
| S54 B | POS | 4:18 |  | POS | 31.29 |  |  |
| S55 A | POS | 11:08 | POS | POS | 33.27 | POS | Y |
| S55 B | POS | 11:56 |  | POS | 22.69 |  |  |
| S56 A | NEG | - | NEG | POS | 30.44 | INC | P INC |
| S56 B | NEG | - |  | NEG | - |  |  |
| S57 A | POS | 5:06 | POS | NEG | - | NEG | N |
| S57 B | POS | 5:09 |  | NEG | - |  |  |
| S58 A | POS | 8:12 | POS | POS | 27.33 | POS | Y |
| S58 B | POS | 7:23 |  | POS | 28 |  |  |
| S59 A | POS | 5:20 | POS | NEG | - | NEG | N |
| S59 B | POS | 4:47 |  | NEG | - |  |  |
| S60 A | POS | 4:41 | POS | POS | 31.57 | POS | Y |
| S60 B | POS | 4:19 |  | POS | 31.37 |  |  |
| S61 A | NEG | - | NEG | POS | 34.7 | POS | N |
| S61 B | NEG | - |  | POS | 35.66 |  |  |
| S62 A | POS | 17:11 | POS | NEG | - | NEG | N |
| S62 B | POS | 17:26 |  | NEG | - |  |  |
| S63 A | NEG | - | NEG | NEG | - | NEG | Y |
| S63 B | NEG | - |  | NEG | - |  |  |
| S64 A | NEG | - | NEG | NEG | - | NEG | Y |
| S64 B | NEG | - |  | NEG | - |  |  |
| S65 A | NEG | - | NEG | NEG | - | NEG | Y |
| S65 B | NEG | - |  | NEG | - |  |  |
| S66 A | NEG | - | NEG | NEG | - | NEG | Y |
| S66 B | NEG | - |  | NEG | - |  |  |
| S67 A | POS | 5:50 | POS | POS | 32.68 | POS | Y |
| S67 B | POS | 5:39 |  | POS | 33.41 |  |  |
| S68 A | NEG | 3:03 | NEG | POS | 30.64 | POS | Y |
| S68 B | NEG | 3:04 |  | POS | 31.97 |  |  |
| S69 A | POS | 4:37 | POS | POS | 33.66 | POS | Y |
| S69 B | POS | 4:35 |  | POS | 31.18 |  |  |
| S70 A | POS | 7:45 | POS | POS | 32.27 | POS | Y |
| S70 B | POS | 7:38 |  | POS | 32.2 |  |  |
| S71 A | POS | 4:16 | POS | POS | 34.33 | POS | Y |
| S71 B | POS | 4:05 |  | POS | 32.3 |  |  |
| S72 A | POS | 17:23 | POS | POS | 32.46 | POS | Y |
| S72 B | POS | 17:12 |  | POS | 31.56 |  |  |
| S73 A | POS | 15:57 | INC | POS | 31.33 | POS | L INC |
| S73 B | NEG | - |  | POS | 33.05 |  |  |
| S74 A | POS | 7:00 | POS | POS | 34.2 | POS | Y |
| S74 B | POS | 6:22 |  | POS | 31.52 |  |  |
| S75 A | POS | 2:46 | POS | POS | 33.45 | POS | Y |
| S75 B | POS | 2:47 |  | POS | 32.85 |  |  |
| S76 A | NEG | 29:38 | NEG | POS | 31.09 | POS | N |
| S76 B | NEG | 25:42 |  | POS | 32.84 |  |  |
| S77 A | NEG | 22:19 | INC | POS | 34.54 | POS | L INC |
| S77 B | POS | 19:21 |  | POS | 33.59 |  |  |
| S78 A | NEG | 21:43 | INC | NEG | - | NEG | L INC |
| S78 B | POS | 19:53 |  | NEG | - |  |  |
| S79 A | POS | 1:50 | INC | POS | 33.49 | POS | L INC |
| S79 B | NEG | - |  | POS | 33.1 |  |  |
| S80 A | POS | 5:11 | POS | POS | 31.15 | POS | Y |
| S80 B | POS | 5:17 |  | POS | 13.86 |  |  |
| S81 A | POS | 6:58 | POS | NEG | - | NEG | N |
| S81 B | POS | 6:03 |  | NEG | - |  |  |
| S82 A | POS | 2:50 | POS | POS | 36.29 | POS | Y |
| S82 B | POS | 2:48 |  | POS | 33.1 |  |  |
| S83 A | POS | 9:49 | POS | NEG | - | NEG | N |
| S83 B | POS | 8:54 |  | NEG | - |  |  |
| S84 A | POS | 2:17 | POS | POS | 35.61 | INC | P INC |
| S84 B | POS | 2:18 |  | NEG | - |  |  |
| S85 A | POS | 5:45 | POS | POS | 31.55 | POS | Y |
| S85 B | POS | 6:36 |  | POS | 33.04 |  |  |
| S86 A | POS | 4:17 | POS | POS | 35.01 | POS | Y |
| S86 B | POS | 4:26 |  | POS | 32.35 |  |  |
| S87 A | NEG | - | NEG | POS | 33.21 | POS | N |
| S87 B | NEG | - |  | POS | 35.96 |  |  |
| S88 A | POS | 18:29 | POS | NEG | - | NEG | N |
| S88 B | POS | 15:21 |  | NEG | - |  |  |
| S89 A | POS | 8:44 | POS | POS | 35.08 | POS | Y |
| S89 B | POS | 7:45 |  | POS | 31.16 |  |  |
| S90 A | NEG | - | NEG | POS | 30.7 | POS | N |
| S90 B | NEG | - |  | POS | 33.36 |  |  |
| S91 A | POS | 4:59 | POS | POS | 33.68 | POS | Y |
| S91 B | POS | 4:23 |  | POS | 33.55 |  |  |
| S92 A | POS | 3:33 | POS | POS | 31.75 | POS | Y |
| S92 B | POS | 3:10 |  | POS | 31.87 |  |  |
| S93 A | POS | 2:28 | POS | NEG | - | INC | P INC |
| S93 B | POS | 2:18 |  | POS | 30.41 |  |  |
| S94 A | POS | 12:25 | POS | POS | 34.08 | POS | Y |
| S94 B | POS | 9:05 |  | POS | 31.15 |  |  |
| S95 A | POS | 7:26 | POS | POS | 33.71 | POS | Y |
| S95 B | POS | 3:20 |  | POS | 30.71 |  |  |
| S96 A | POS | 1:47 | POS | POS | 31.6 | POS | Y |
| S96 B | POS | 2:39 |  | POS | 31.35 |  |  |
| S97 A | POS | 12:13 | POS | POS | 31.82 | POS | Y |
| S97 B | POS | 10:08 |  | POS | 31.25 |  |  |
| S98 A | NEG | - | NEG | POS | 32.84 | POS | N |
| S98 B | NEG | - |  | POS | 26.07 |  |  |
| S99 A | POS | 5:31 | POS | POS | 33.32 | POS | Y |
| S99 B | POS | 5:16 |  | POS | 33.37 |  |  |
| S100 A | POS | 6:02 | POS | POS | 32.71 | POS | Y |
| S100 B | POS | 6:16 |  | POS | 31.46 |  |  |
| S101 A | POS | 4:37 | POS | POS | 33.17 | POS | Y |
| S101 B | POS | 5:04 |  | POS | 31.8 |  |  |
| S102 A | POS | 4:17 | POS | POS | 32.26 | POS | Y |
| S102 B | POS | 4:00 |  | POS | 33.1 |  |  |
